# Prosecution of non-disclosure of HIV status: Potential impact on HIV testing and transmission among HIV-negative men who have sex with men

**DOI:** 10.1371/journal.pone.0193269

**Published:** 2018-02-28

**Authors:** Maya A. Kesler, Rupert Kaul, Mona Loutfy, Ted Myers, Jason Brunetta, Robert S. Remis, Dionne Gesink

**Affiliations:** 1 Department of Epidemiology, Dalla Lana School of Public Health, University of Toronto, Toronto, Ontario, Canada; 2 Department of Medicine, University Health Network, University of Toronto, Toronto, Ontario, Canada; 3 Women’s College Hospital, Toronto, Ontario, Canada; 4 Maple Leaf Medical Clinic, Toronto, Ontario, Canada; University of New South Wales, AUSTRALIA

## Abstract

**Background:**

Non-disclosure criminal prosecutions among gay, bisexual and other men who have sex with men (MSM) are increasing, even though transmission risk is low when effective antiretroviral treatment (ART) is used. Reduced HIV testing may reduce the impact of HIV “test and treat” strategies. We aimed to quantify the potential impact of non-disclosure prosecutions on HIV testing and transmission among MSM.

**Methods:**

MSM attending an HIV and primary care clinic in Toronto completed an audio computer-assisted self-interview questionnaire. HIV-negative participants were asked concern over non-disclosure prosecution altered their likelihood of HIV testing. Responses were characterized using cross-tabulations and bivariate logistic regressions. Flow charts modelled how changes in HIV testing behaviour impacted HIV transmission rates controlling for ART use, condom use and HIV status disclosure.

**Results:**

150 HIV-negative MSM were recruited September 2010 to June 2012. 7% (9/124) were less or much less likely to be tested for HIV due to concern over future prosecution. Bivariate regression showed no obvious socio/sexual demographic characteristics associated with decreased willingness of HIV testing to due concern about prosecution. Subsequent models estimated that this 7% reduction in testing could cause an 18.5% increase in community HIV transmission, 73% of which was driven by the failure of HIV-positive but undiagnosed MSM to access care and reduce HIV transmission risk by using ART.

**Conclusions:**

Fear of prosecution over HIV non-disclosure was reported to reduce HIV testing willingness by a minority of HIV-negative MSM in Toronto; however, this reduction has the potential to significantly increase HIV transmission at the community level which has important public health implications.

## Introduction

In Canada, between January 1989 and December 2015, 181 HIV-positive persons were criminally charged for failing to disclose their HIV status prior to sex [1]. Almost half of HIV non-disclosure criminal cases have occurred in Ontario with aggravated sexual assault being the most common charge [2]. Historically, the majority of these charges were filed against men engaging in heterosexual sex; however, criminal cases involving defendants who are gay, bisexual or other men who have sex with men (MSM) has recently increased, to where between 2011 and 2015, 34% of men charged were MSM [1–3].

In the United States (U.S.), HIV criminalization laws are dictated by the State and are not consistent State by State [4]. The most common type of U.S. State HIV criminalization law is one that requires HIV-positive individuals to disclose their HIV-positive status to sexual partners [4]. Prior to 1998, there was no Canadian specific Federal Supreme Court of Canada (SCC) ruling that addressed disclosure of HIV status [5]. However, in 1998, the SCC ruled that HIV-infected persons had a legal duty to disclose their status before engaging in sexual acts that posed a “significant risk of serious bodily harm” [6]. However, “significant risk” was poorly defined and led to inconsistencies in lower Court rulings [5]. In 2012, the SCC revisited the matter and established that people living with HIV must disclose prior to sex acts that pose a “realistic probability” of HIV transmission [7, 8]. When defining this new standard, the SCC found that there is a duty to disclose before vaginal sex unless a condom is used and the accused’s viral load at the time of sexual relations is “low” (e.g. less than 1,500 copies per milliliter) [7, 8]. HIV transmission is not required to have occurred for criminal charges to be laid or for a guilty verdict [2]. To date, Canada has one of the strictest HIV criminalization laws worldwide [9].

The impact of the fear of prosecution on HIV testing in HIV-negative MSM is unclear. There are concerns that in HIV-negative persons, HIV criminalization laws may deter persons from HIV testing, since not knowing their HIV status may eliminate the risk of being charged with an HIV related criminal offense [10–14]. There are several negative consequences to decreased HIV testing. Treatment as prevention (TasP) (treating HIV-positive individuals with anti-HIV drugs) has been shown to be greatly effective at reducing HIV transmission [15–19]. Preliminary and follow-up results of two MSM studies where serodiscordant couples engaged in condomless anal sex when the HIV-positive partner was on suppressive ART found no HIV seroconversions, even when the HIV-negative individual was the receptive partner and ejaculation occurred [17, 18]. However, treatment and engagement in the care cascade can only begin among individuals who test for HIV which is why the Ontario Advisory Committee on HIV/AIDS (OACHA) group expanded the “treatment cascade” to include HIV prevention, engagement and care [20]. If HIV criminalization laws have the potential to deter HIV testing, even in small numbers, there is a potential for a pool of individuals to be created who are unaware of their HIV status. This, in turn, may reduce the pool of HIV-positive individuals on ART which increases the number of HIV-positive individuals with lower CD4 counts and uncontrolled/high viral loads. This would be in direct contravention to the OACHA 2026 goals which include increasing HIV testing and early HIV diagnosis, fast linkage to care and increasing the support given to people living with HIV [20].

There are a growing number of HIV criminalization cases involving MSM in Canada [1–3] and little information about whether or how HIV criminalization laws affect HIV testing behaviour. The aims of our study were to: 1) determine if self-reported HIV-negative MSM were more or less likely to get an HIV test due to concern about prosecution and what, if any, socio-sexual behaviour characteristics were associated with this and 2) estimate the impact decreased HIV testing could have on HIV transmission rates.

## Methods

### Study setting and eligibility

Data were derived from the HIV/STI Co-Infections Study and has been described elsewhere [21, 22]. Briefly, the overall study recruited HIV-negative and HIV-positive MSM through the Maple Leaf Medical Clinic (MLMC), a large primary care clinic in Toronto, from September 2010 to June 2012. Recruitment occurred leading up to the 2012 HIV criminalization SCC clarification decision on what was required with respect to HIV status disclosure. This data is important as it gives context as to what sexual health decision making was occurring among MSM during a time of uncertainty within the law. Using this data from before the new 2012 SCC decision will also enable researchers to use this as “before” data and compare this to data that is becoming available from after the SCC decision.” Participants were eligible if they were male, 16 years or older, had sex with another man in the previous 12 months, and lived in the Greater Toronto Area. This analysis was restricted to HIV-negative participants.

### Data collection

After eligibility criteria were met and consent was obtained, participants completed a self-administered questionnaire using ACASI (Audio Computer Assisted Self-Interview; Questionnaire Development System (QDS) Version 2.5, Nova Research Company, Bethesda, Maryland, USA). Survey questions included demographic and sexual behaviour characteristics. Participants also provided blood, urine and anal swab samples to confirm self-reported HIV status and test for sexually transmitted infections (STIs). Results from serologic testing were reported to participants at a later date by either a health official (for a reportable disease) or at their next clinic visit. Participants were compensated $50 for their time, travel, knowledge and biologic samples. Participants answered the questionnaire after agreeing to STI/HIV testing but before they received any serologic test results. Data was de-identified for the analysis and any identifiable data was destroyed three months after recruitment was completed.

### Ascertainment of the outcome

The outcome variable was whether concerns about being prosecuted prevented HIV-negative MSM from being tested for HIV. The survey provided the following background information: “In Ontario, some HIV-positive persons have been prosecuted (put on trial) for having sex without disclosing their HIV status.” Participants were then asked: “How much would a concern about being prosecuted affect your decision to get tested for HIV?” Participants answered on a 5-point Likert scale that ranged from much less likely to much more likely to be tested for HIV. For cross tabulations, the variable had three categories: more and much more likely, neutral, and less or much less likely. This allowed for a determination of whether the neutral category was different from the more or less likely categories. Bivariate logistic regression was then performed and the variable was dichotomized into less likely (much less likely and less likely) and no change/more likely (neutral, more likely, much more likely). This was done since being less or much less likely to get an HIV test is a negative event, whereas neutral or more or much more likely is not a negative event.

### HIV transmission potential flow charts

**Fig 1:**
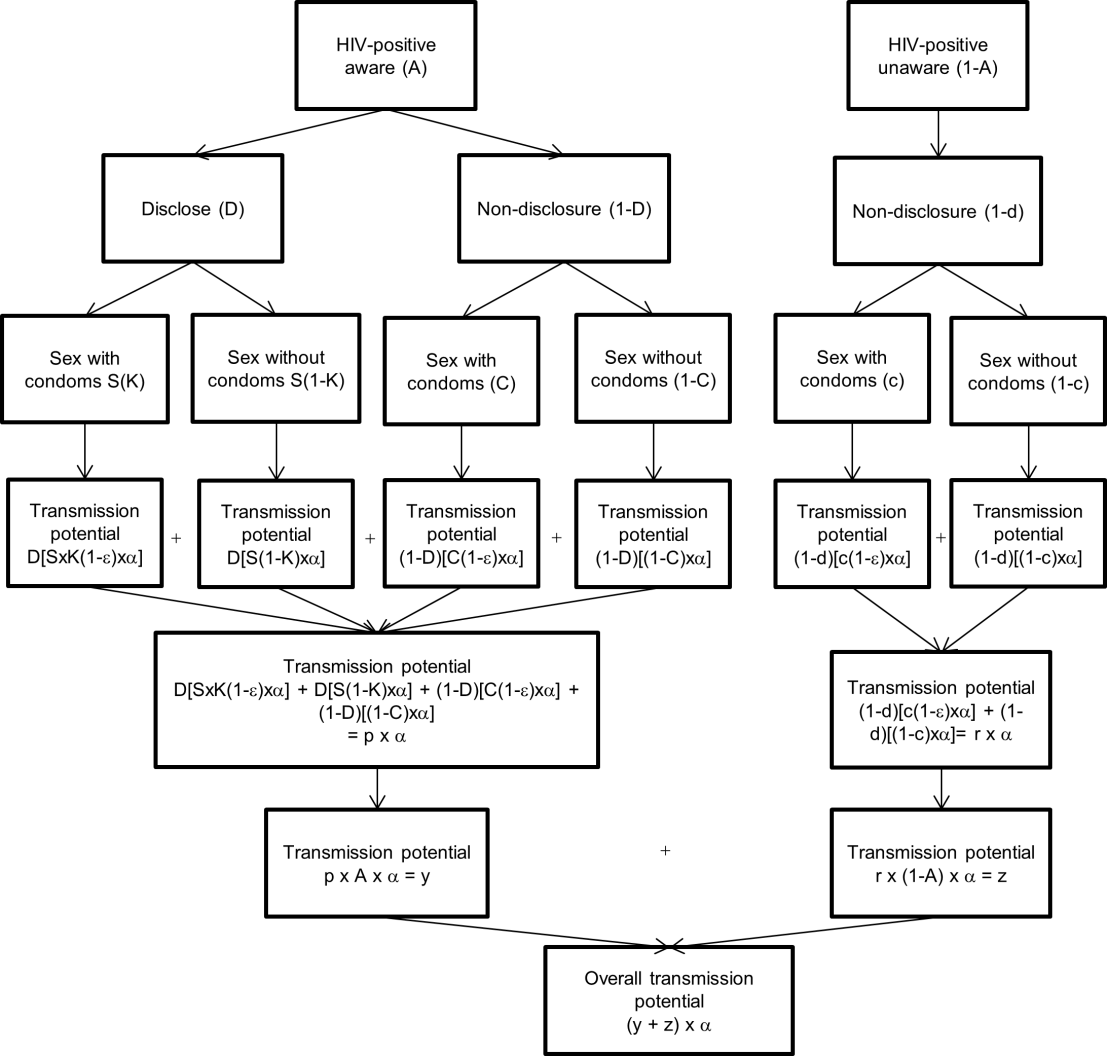
HIV transmission potential not taking into account antiretroviral therapy (ART) use among men who have sex with men (MSM) engaging in anal sex. **Constant values throughout analysis:** D- Proportion who disclose among those who are aware of their status (71.86%) S- Proportion who agree to sex after disclosure among those who are aware of their status (69.90%) K- Proportion who use a condom after disclosure among those aware of their status (70.90%) C- Proportion who use a condom if no disclosure among those aware of their status (60%) d- Proportion who disclose among those who do not know their status (0%) c- Proportion who use a condom if no disclosure among those who do not know their status (53%) ε- Condom effectiveness during anal intercourse (70%) α- Probability of transmission through unprotected anal intercourse **Non-constant values throughout analysis:** A-HIV-positive aware.

**Fig 2:**
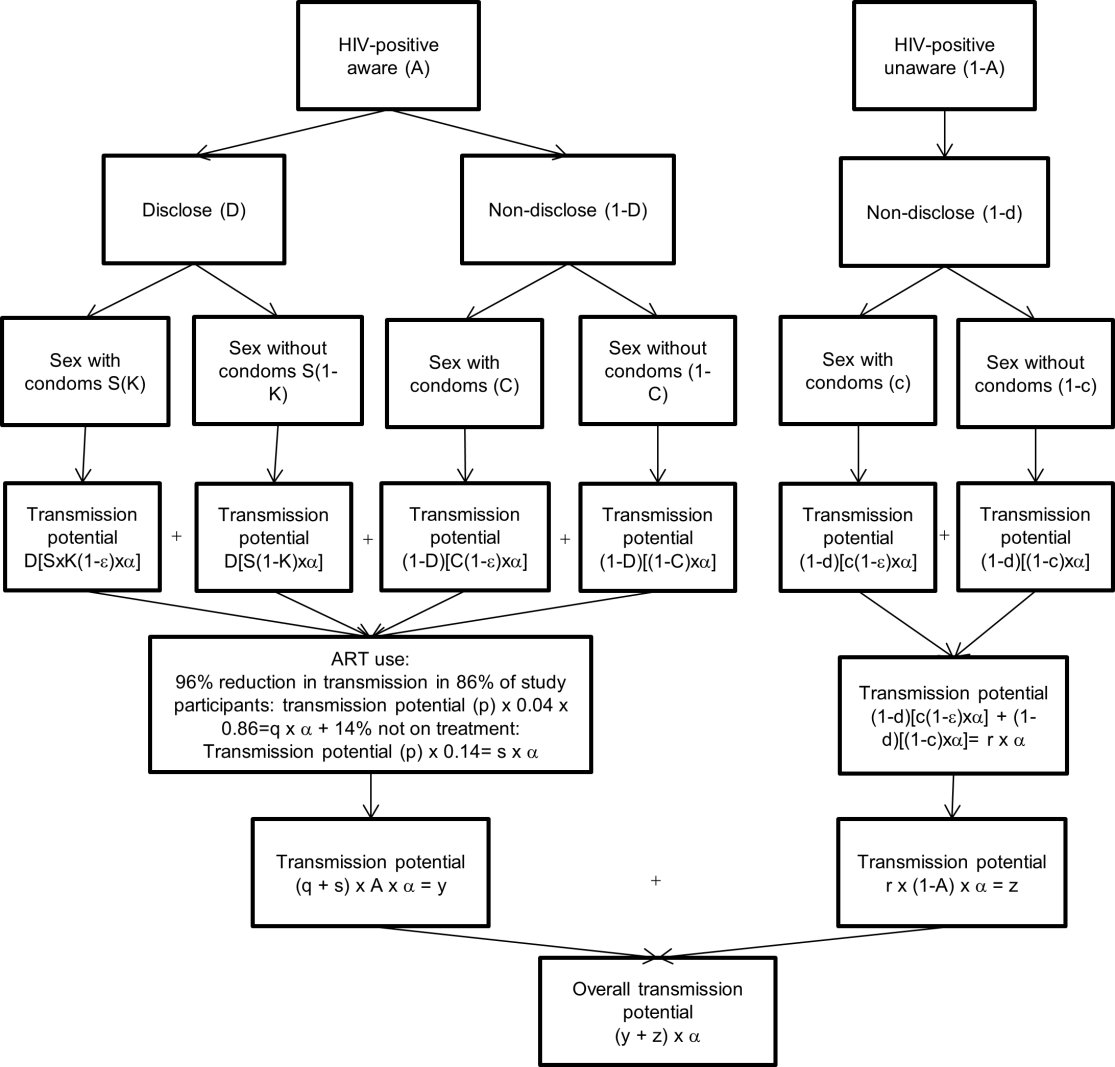
HIV transmission potential taking into account antiretroviral therapy (ART) use among men who have sex with men (MSM) engaging in anal sex. Where p = D[SxK(1-ε)xα] + D[S(1-K)xα] + (1-D)[C(1-ε)xα] + (1-D)[(1-C)xα] **Constant values throughout analysis:** D- Proportion who disclose among those who are aware of their status (71.86%) S- Proportion who agree to sex after disclosure among those who are aware of their status (69.90%) K- Proportion who use a condom after disclosure among those aware of their status (70.90%) C- Proportion who use a condom if no disclosure among those aware of their status (60%) d- Proportion who disclose among those who do not know their status (0%) c- Proportion who use a condom if no disclosure among those who do not know their status (53%) ε- Condom effectiveness during anal intercourse (70%) α- Probability of transmission through unprotected anal intercourse **Non-constant values throughout analysis:** A-HIV-positive aware.

The tree diagram for HIV transmission created by O’Connell, Reed and Serovich [23] was originally based on the mathematical modeling methods of Pinkerton and Galletly [24] to determine the efficacy of serostatus disclosure and condom use on HIV transmission risk. We used these tree diagrams as the basis for a flow chart to estimate the probability of HIV transmission under multiple different modeling scenarios. This included determining the contribution that HIV-positive aware and HIV-positive unaware individuals have on HIV transmission rates. Disclosure rates, condom rates with and without disclosure, condom effectiveness and probability of engaging in sex after disclosure remained constant in our flow charts. The first flow chart describes HIV transmission potential not taking into account antiretroviral therapy (ART) use (Fig 1) while the second flow chart describes HIV transmission potential when taking into account ART use (Fig 2).

We parameterized our flow charts using our observed study numbers when possible so our estimates of HIV transmission were specific to our study population. The mathematical model is based on empirical evidence from our study where possible, and from other Canadian and U.S. studies where this data was not available in our study. An HIV-positive aware individual was defined as having tested positive for HIV and being aware of their positive status [P(aware) = A = 82%]. An HIV-positive unaware individual was defined as an HIV-positive individual who either had not been tested for HIV, or had been tested but not made aware of the results [P(unaware) = A-1 = 18%]. These proportions were taken from the Canadian national average among MSM who are unaware of their HIV-positive status [25]. Similar to O’Connell et al [23], the following conventions were used among HIV-positive aware MSM: proportion in our study who disclosed their positive status to casual or regular male partners at the time of their HIV diagnosis [P(disclosure) = D = 71.86%], proportion who engage in intercourse after disclosure [P(agrees to intercourse | disclosure = S = 69.9%)], proportion who used condoms after disclosure [P(condom use | intercourse after disclosure) = K = 70.9%], proportion who used condoms given no disclosure [P(condom use | intercourse without disclosure) = C = 60%]. S, K and C were not available in our study so estimates given by O’Connell et al were used [23]. Condom effectiveness (ε) in MSM during anal sex was set at 70%, as found by Smith, Herbst, Zhang and Rose [26]. Among HIV-positive but unaware MSM, non-disclosure was 100% because individuals cannot disclose if they don’t know their status [P(non-disclosure = 1-d = 100%] and condom use proportions [P(condom use) = c = 53%] were taken from HIV-negative study participants because HIV-positive unaware MSM were theorized to engage in behaviour similar to individuals who believed they were HIV-negative. The proportions used in the flow charts and their assigned representative ‘letter’ is summarized in Table 1.

**Table 1:** Definitions, corresponding proportions and ‘letter’ assigned for HIV transmission flow charts (Figs 1 and 2).

Letter	Definition	Proportions
A	Aware of HIV-positive status	82.0%^a^
1-A	Not aware of HIV-positive status	18.0%^a^
ε	Condom effectiveness during anal sex	70.0%^b^
D	HIV status disclosure among HIV-positive aware	71.9%^c^
1-D	No HIV status disclosure among HIV-positive aware	28.1%^c^
S	Agree to sex after HIV-positive status disclosure	69.9%^c^
K	Condom use after HIV-positive status disclosure	70.9%^c^
1-K	No condom use after HIV-positive status disclosure	29.1%^c^
C	Condom use without HIV status disclosure	60.0%
1-C	No condom use without HIV status disclosure	40.0%
d	HIV status disclosure among HIV-positive unaware	0.0%
1-d	No HIV status disclosure among HIV-positive unaware	100.0%
c	Condom use among HIV-positive unaware	53.0%
1-c	No condom use among HIV-positive unaware	47.0%
p	Transmission potential among HIV-positive aware individuals given condom use and disclosure proportions	
q	Transmission potential among HIV-positive aware individuals on ART	
s	Transmission potential among HIV-positive aware individuals not on ART	
y	Transmission potential among HIV-positive aware individuals given they make up 'A' proportion of HIV-positive population	
r	Transmission potential among HIV-positive unaware individuals given condom use and disclosure proportions	
z	Transmission potential among HIV-positive unaware individuals given they make up '1-A' proportion of HIV-positive population	
y+z	Overall transmission potential among HIV-positive individuals	
α	Probability of HIV transmission through unprotected anal intercourse	

All proportions taken from study unless otherwise specified by reference letter.

Reference

^a^:[25]

^b^:[26]

^c^:[23]

The baseline flow chart (model 1) represents the effect of HIV testing as a probability of transmission after controlling for disclosure, condom use, condom effectiveness and agreeing to sex after disclosure without taking into account ART use. We then decreased the number of HIV-positive aware individuals (82% in model 1) by the proportion who said they were less likely to get an HIV test due to concern about being prosecuted (7% of 82%), not taking into account ART use (model 2). This model assumed that all individuals who would be less likely to test would go from the ‘HIV-positive aware’ category, to the ‘HIV-positive unaware’ category. Hence, the ‘HIV-positive unaware’ category increased from the baseline of 18% to 23.7% in model 2. Models 1 and 2 are based on the transmission potential described by Fig 1. Model 3 used baseline numbers, but factored in ART use by HIV-positive aware individuals to estimate the contribution of ART use in decreasing HIV transmission probability. It assumed that 86% of HIV-positive aware individuals were on ART (as reported in our study) and assumed 96% effectiveness of ART [15]. Model 4 used model 3 (taking into account ART use) as the baseline model, but decreased the proportion of individuals tested for HIV (same as in model 2) and hence, increased the number of HIV-positive unaware individuals (by the same percentage in model 2). Models 5 and 6 were the same as model 4, but decreased the number of individuals going from the HIV-positive aware category to the HIV-positive unaware category by smaller proportions (18% x the proportion found to be less likely to test for HIV (7%) x 82% HIV-positive aware for model 6 and 50% x the proportion found to be less likely to test for HIV (7%) x 82% HIV-positive aware for model 6). Model 7 used Model 3 as the baseline model but modified ART effectiveness from 96% down to 76%, to simulate decreased ART effectiveness, and hence, less individuals reaching viral suppression [27]. Models 3–7 are based on the transmission potential described by Fig 2.

### Statistical analysis

Data were analyzed using SAS 9.3 (SAS Institute, Cary, NC) and STATA 13 (StataCorp. 2013. *Stata Statistical Software*: *Release 13*. College Station, TX: StataCorp LP). Demographic and sexual behaviour characteristics of participants were described using median and inter-quartile ranges for continuous variables and proportions for categorical variables. Pearson’s Chi-square tests for cross tabulations and odds ratios (OR) and 95% confidence intervals (CI) for bivariate logistic regression were reported to assess the relationship between socio-demographic and sexual behaviour characteristics and HIV testing due to concern about prosecution. Multivariable logistic regression was not possible due to low variability. Ethics approval was obtained from the University of Toronto REB.

## Results

### Study population

A total of 150 HIV-negative MSM were recruited, consented to participate, completed the questionnaire and provided biological samples. The median age was 44.5 years (IQR 37–50 years) (Table 2). The majority of participants were White and had at least some undergraduate education. Over one quarter had used amyl nitrites (poppers) in the previous six months, and almost 10% had used methamphetamines in the previous six months. Almost 50% were single and never married, and almost 75% had been diagnosed with an STI in their lifetime.

**Table 2:** Demographic and other characteristics among HIV-negative MSM.

Characteristic		n (%)
		n = 150
Age (years)	Median (IQR)	44.5 (37–50)
Age (years)	<30	10 (6.76)
	30–39	39 (26.35)
	40–49	58 (39.19)
	50–59	32 (21.62)
	60+	9 (6.08)
Ethnicity	White	121 (82.88)
	Other	25 (17.12)
Education	High School or less	11 (7.33)
	Some or completed undergrad	106 (70.67)
	Some or completed grad	33 (22.00)
Personal Income	$19,999 or less	36 (24.32)
	$20,000-$59,999	63 (42.57)
	$60,000-$99,999	31 (20.95)
	$100,000 or more	18 (12.16)
Used amyl nitrite (poppers) in previous 6 months	No	107 (72.30)
	Yes	41 (27.70)
Used methamphetamines or crystal meth in previous 6 months	No	134 (90.54)
	Yes	14 (9.46)
Number of casual partners, previous 6 months	None	50 (33.78)
	1	9 (6.08)
	2–4	28 (18.92)
	5–9	20 (13.51)
	10 or more	41 (27.71)
Number of regular partners, previous 6 months	None	30 (20.13)
	1	69 (46.31)
	2–4	36 (24.16)
	5 or more	14 (9.40)
Ever STI diagnosis	No	42 (28.00)
	Yes	108 (72.00)
Marital status	Married/common law to male or female	56 (37.58)
	Divorced/separated/ widowed from male or female	24 (16.11)
	Single, never married	69 (46.31)

MSM: men who have sex with men; IQR: Interquartile range; STI: sexually transmitted infection

### Covariates of HIV testing due to concern about prosecution among HIV-negative MSM

One hundred and twenty-nine HIV-negative participants answered the question about a concern about being prosecuted affecting HIV-testing decisions. Seven (5.4%) were much less likely to get tested, two (1.6%) were less likely to get tested, 90 (69.8%) reported no change, 11 (8.5%) were more likely to get tested, and 14 (10.9%) were much more likely to get tested. Five (3.9%) refused to answer or reported ‘don’t know’. Therefore, a total of 7% (9/124) were less likely to get an HIV test due to concern about prosecution (SD .023, 95% CI 2.69%-11.82%).

Due to low variability, only cross-tabulations and bivariate regressions were possible. There were no obvious patterns or groupings that differentiated those participants who were more, neutral, or less likely to get HIV tested due to concern about prosecution (Table 3). Among those who were less likely to get tested due to concern about prosecution, the majority were White, had a higher education and higher personal income and did not use poppers or methamphetamines. There was no trend among sexual behaviour characteristics and being less likely to get HIV tested due to concern about prosecution. Using bivariate logistic regression, there were no statistically significant characteristics found to be associated with being less likely to get an HIV test due to concern about prosecution.

**Table 3:** Characteristics of HIV-negative MSM and their associations with HIV testing due to concern about prosecution.

Characteristic		More or much more likely to get tested (n = 25)	No change in likelihood of HIV testing (n = 90)	Less or much less likely to get tested (n = 9)	Unadjusted OR* (95% CI)
		n (%)	n (%)	n (%)	
Age (years)	Continuous (median, IQR)	43 (37.5–47)	44 (36–51)	45 (33–51)	1.00 (0.94–1.07)
Age (years)	<30	3 (12.50)	5 (5.62)	2 (22.22)	1.00
	30–39	6 (25.00)	26 (29.21)	1 (11.11)	0.13 (0.010–1.56)
	40–49	11 (45.83)	32 (35.96)	3 (33.33)	0.28 (0.040–1.95)
	50–59	3 (12.50)	21 (23.60)	2 (22.22)	0.33 (0.040–2.77)
	60+	1 (4.17)	5 (5.62)	1 (11.11)	0.67 (0.048–9.19)
Ethnicity	White	18 (72.00)	76 (87.36)	7 (77.78)	1.00
	Other	7 (28.00)	11 (12.64)	2 (22.22)	1.49 (0.29–7.78)
Education	Some undergrad or less	8 (32.00)	23 (25.56)	3 (33.33)	1.00
	Completed undergrad or more	17 (68.00)	67 (74.44)	6 (72.58)	0.74 (0.17–3.13)
Personal Income	$19,999 or less	6 (24.00)	21 (23.86)	1 (11.11)	1.00
	$20,000-$39,999	7 (28.00)	14 (15.91)	2 (22.22)	2.57 (0.22–30.32)
	$40,000 or more	12 (48.00)	53 (60.23)	6 (66.67)	2.49 (0.29–21.70)
Used amyl nitrite (poppers) in previous 6 months	No	19 (79.17)	61 (68.54)	8 (88.89)	1.00
	Yes	5 (20.83)	28 (31.46)	1 (11.11)	0.30 (0.036–2.52)
Used methamphetamines or crystal meth in previous 6 months	No	23 (95.83)	79 (88.76)	7 (77.78)	1.00
	Yes	1 (4.17)	10 (11.24)	2 (22.22)	2.65 (0.49–14.36)
Number of casual partners, previous 6 months	None	11 (45.83)	27 (30.34)	4 (44.44)	1.00
	1–4	6 (25.00)	21 (23.60)	2 (22.22)	0.70 (0.12–4.12)
	5–9	3 (12.50)	13 (14.61)	2 (22.22)	1.19 (0.20–7.15)
	10 or more	4 (16.67)	28 (31.46)	1 (11.11)	0.30 (0.032–2.79)
Number of regular partners, previous 6 months	None	5 (20.00)	17 (19.10)	1 (11.11)	1.00
	1–4	18 (72.00)	64 (71.91)	6 (66.67)	1.61 (0.18–14.08)
	5 or more	2 (8.00)	8 (8.99)	2 (22.22)	4.40 (0.36–54.37)
Ever STI diagnosis	No	7 (28.00)	23 (25.56)	4 (44.44)	1.00
	Yes	18 (72.00)	67 (74.44)	5 (55.56)	0.44 (0.11–1.75)
Marital status	Married/common law to male or female	11 (44.00)	33 (37.08)	3 (33.33)	1.00
	Divorced/separated/ widowed from male or female	4 (16.00)	14 (15.73)	2 (22.22)	1.63 (0.25–10.59)
	Single, never married	10 (40.00)	42 (47.19)	4 (44.44)	1.13 (0.24–5.31)
Chance of HIV infection	Low	20 (83.33)	69 (79.31)	8 (88.89)	1.00
	High	4 (16.67)	18 (20.69)	1 (11.11)	0.51 (0.060–4.26)
Condom use during insertive anal sex with casual male partner**	Always	4 (50.00%)	13 (37.14%)	1 (33.33%)	1.00
	Sometimes	3 (37.50%)	19 (54.29%)	1 (33.33%)	0.77 (0.05–13.27)
	Never	1 (12.50%)	3 (8.57%)	1 (33.33%)	4.25 (0.22–83.52)
Condom use during receptive anal sex with casual male partner**	Always	7 (70.00%)	19 (54.29%)	0 (0%)	†
	Sometimes	2 (20.00%)	13 (37.14%)	0 (0%)	
	Never	1 (10.00%)	3 (8.57%)	1 (100%)	
Condom use during insertive anal sex with an HIV-positive regular male partner**	Always	1 (100%)	2 (18.18%)	0 (0%)	†
	Sometimes	0 (0%)	3 (27.27%)	0 (0%)	
	Never	0 (0%)	6 (54.55%)	0 (0%)	
Condom use during receptive anal sex with an HIV-positive regular male partner**	Always	1 (100%)	4 (36.36%)	0 (0%)	†
	Sometimes	0 (0%)	4 (36.36%)	0 (0%)	
	Never	0 (0%)	3 (27.27%)	1 (100%)	
Condom use during insertive anal sex with an HIV-unknown status regular male partner**	Always	4 (66.67%)	4 (44.44%)	1 (100%)	†
	Sometimes	2 (33.33%)	2 (22.22%)	0 (0%)	
	Never	0 (0%)	3 (33.33%)	0 (0%)	
Condom use during receptive anal sex with an HIV-unknown status regular male partner**	Always	4 (80.00%)	2 (28.57%)	0 (0%)	†
	Sometimes	1 (20.00%)	2 (28.57%)	1 (100%)	
	Never	0 (0%)	3 (42.86%)	0 (0%)	
Year of survey	2011	11 (44.00)	45 (50.00)	7 (77.78)	1.00
	2012	14 (56.00)	45 (50.00)	2 (22.22)	0.27 (0.054–1.36)

*Note unadjusted OR is comparison between binary categories of much more likely, more likely and neutral versus less likely and much less likely to get HIV tested due to concern about prosecution

** Condom use only among those engaging in that sexual behaviour

† Bivariate logistic regression unable to be performed due to low variability of the outcome

### HIV transmission potential

We found that the 18% that makes up the HIV-positive but unaware group contributed 25% to the overall transmission potential when not taking into account ART use (model 1) (Table 4). In model 2, we found that the 23.7% that makes up the HIV-positive unaware group would account for 32% of the total transmission potential if HIV testing decreased by 7% and assuming all 7% were HIV-positive but unaware. When ART was introduced into the model (model 3), with the assumption that 86% of the HIV-positive population was on ART (as was with our study) and 96% ART effectiveness, there was a significant decrease in overall transmission potential. However, the 18% that makes up the HIV-positive but unaware group now accounted for 66% (model 3) compared to 25% (model 1) of the total HIV transmission potential. In model 4, if HIV testing decreased by 7%, assuming all non-testers were HIV-positive, while taking into account ART use, the 24% that makes up the HIV-positive but unaware group would account for 73% of the total transmission potential. This 7% decrease in HIV testing would result in an 18.5% increase in overall HIV transmission potential. In model 5, with only 18% of the 7% who were less likely to get HIV tested remaining HIV-positive but unaware, the 19.0% that make up the HIV-positive but unaware group accounted for 67% of the total HIV transmission potential. This 1.0% decrease in HIV testing would result in a 3.4% increase in overall HIV transmission potential. In model 6, the 20.9% who would remain HIV-positive unaware accounted for 70% of the overall transmission potential. The 2.9% decrease in HIV testing would result in a 9.4% increase in overall HIV transmission potential. In the final model (model 7) where ART effectiveness decreased to 76%, the 18% that make up the HIV-positive unaware group accounted for 49% of the HIV transmission potential.

**Table 4:** HIV transmission potential among HIV-negative aware and unaware MSM.

				Transmission Potential			Proportion of total transmission potential	
		HIV-positive aware (%)	HIV-positive unaware (%)	Overall	HIV-positive aware	HIV-positive unaware	HIV-positive aware	HIV-positive unaware
Corresponding letter to flow chart		A	1-A	y + z	y	z	y / y + z	z / y+ z
Model 1								
	Baseline; no ART use in the model	82.0%	18.0%	0.4546	0.3414	0.1132	75.1%	24.9%
Model 2								
	7% decrease in testing x 82% = 5.7% increase in HIV-positive unaware; no ART use in model	76.3%	23.7%	0.4668	0.3175	0.1493	68.0%	32.0%
Model 3								
	Baseline; 86% ART use included in model	82.0%	18.0%	0.1727	0.05950	0.1132	34.5%	65.5%
Model 4								
	7% decrease in testing x 82% = 5.7% increase in HIV-positive unaware; 86% ART use included in model	76.3%	23.7%	0.2047	0.05536	0.1493	27.0%	72.9%
Sensitivity analyses								
Model 5								
	Baseline model 4, 18% of 7% x 82% = 1.0% increase in HIV-positive unaware	81.0%	19.0%	0.1785	0.05878	0.1997	32.9%	67.1%
Model 6								
	Baseline model 4, 50% of 7% x 82% = 2.9% increase in HIV-positive unaware	79.1%	20.9%	0.1889	0.0574	0.1315	30.1%	69.6%
Model 7								
	Baseline model 4, ART effectiveness decreased to 76.2%	82.0%	18.0%	0.2308	0.1176	0.1132	50.9%	49.1%

## Discussion

We found that 7% of HIV-negative MSM in Toronto reported being less likely to undergo HIV testing, fearing prosecution. One study in Canada found 17% of those MSM who were aware of non-disclosure laws in Ottawa, reported that it affected their willingness to get tested for HIV [14]. Another Canadian study among MSM found that 18% agreed or strongly agreed that it was better not to know their HIV status under current legal contexts (recruitment 2011–2012) [28]. They also found that 48% agreed or strongly agreed that criminal prosecutions could deter or stop individuals who think they are HIV-positive from getting HIV tested [28]. It is likely our 7% estimate of HIV-negative participants who responded being less likely to get an HIV test due to fear of prosecution was underestimated because recruitment for this study required that participants accept an HIV test to confirm self-reported HIV status (results given during post-test counseling at a later date). Individuals for whom fear of prosecution was strong enough to be deterred from HIV testing likely refused to participate in our study and/or were less likely to have been in a clinic based setting, where recruitment occurred. Nevertheless, even this possibly underestimated proportion of individuals less likely to get an HIV test due to fear of prosecution had a large impact on community HIV transmission potential. We estimated that in light of ART use by HIV-positive aware individuals, a 7% decrease in HIV testing increased the overall HIV transmission potential by 18.5% and that the majority (73%) of HIV transmission was driven by the unmet needs of HIV-positive unaware individuals.

Our flow chart models were based on the tree diagrams created by the O’Connell, Reed and Serovich [23]. We did, however, build in some important additions. First, we used an updated and MSM anal sex specific condom effectiveness estimate since condom failure is more common during anal versus vaginal sex [26, 29, 30]. Second, the original tree diagram required HIV-positive individuals to be aware of their HIV status. However, HIV-positive but unaware individuals cannot disclose their HIV-positive status or make condom use decisions based on their undiagnosed positive status and are not on ART or monitoring their viral load. This is why HIV-positive but unaware individuals contribute disproportionately to HIV transmission [31–34]. We therefore added a second branch to the tree diagram which represented HIV-positive but unaware individuals. Differentiating between HIV transmission that occurs among HIV-positive aware and unaware individuals is critically important because prevention and intervention campaigns can vary based on target populations (e.g. viral load monitoring for HIV-positive aware individuals versus HIV testing among individuals who believe they are HIV-negative or who are unsure of their HIV status). This also enabled us to modify the proportion of HIV-positive aware and unaware individuals, providing evidence to the negative consequences of decreased HIV testing in light of laws that criminalize HIV non-disclosure. Lastly, O’Connell et al. did not include HIV viral load levels into the tree diagram because of the non-significant chi square tests of association found between the participant’s viral load and their sexual and/or disclosure behaviour. They did, however, suggest that future studies look into ART adherence and viral load levels. Our model did incorporate ART use by HIV-positive aware individuals. We first assumed that ART effectiveness was 96% among all participants on ART [15]. We then modified ART effectiveness down to 76%, to simulate ART effectiveness only among individuals who reached viral suppression [27]. Even after reducing the effectiveness of ART in our model, the 18% of HIV-positive unaware group still contributed a disproportionate amount (49%) to the HIV transmission potential indicating how important ART use is at decreasing HIV transmission.

There is a suggestion from the literature that individuals who are less likely to test for HIV are also engaging in more risky sexual behaviour [14]. One study among Canadian MSM found that those who were less likely to get an HIV test due to HIV criminalization prosecutions also reported a higher number of sex partners in the previous two months [14]. Furthermore, among individuals who reported being HIV-negative or unsure of their status, those less willing to get an HIV test due to HIV criminalization prosecutions were more likely to have never previously had an STI or HIV test [14]. The result of this could be that those individuals who are less likely to get tested due to fear of prosecution are also engaging in more risk, and more likely to keep engaging in more risk, and hence are more likely to be HIV-positive but unaware. Our study assumed that all 7% of individuals who were less likely to get tested for HIV were HIV-positive in the flow chart analysis (going from HIV-positive aware to HIV-positive unaware categories) and this may be an overestimation. We therefore ran two sensitivity analyses which took into account that not all 7% of individuals who were less likely to test would be HIV-positive. First we assumed that only 18% would remain HIV-positive unaware, which reflects the Canadian national average of MSM who are unaware of their HIV-positive status [25]. We then assumed that 50% would remain HIV-positive unaware, which reflects the fact that these individuals may be engaging in riskier behaviour compared to MSM who are not deterred from HIV testing. Although this makes for a less dramatic increase in HIV transmission potential among the HIV-positive unaware group, it is still substantial and still emphasizes the significant contribution that HIV-positive unaware individuals make to overall HIV transmission rates.

Creating a larger pool of HIV-positive unaware individuals by decreasing HIV testing due to fear of prosecution greatly increases the overall HIV transmission potential. Thus, laws that criminalize HIV non-disclosure increase the potential for HIV transmission. The only model in which these laws could indirectly decrease HIV transmission is where condom use would significantly increase among HIV-positive aware and unaware individuals. Most notable, the increase would have to be within the HIV-positive unaware group, as in Canada, the majority of HIV-positive aware MSM are on treatment, and of those on ART treatment, attaining viral suppression is high [27, 35, 36]. Proponents of criminalization laws assert that the goal is to protect the sexual partners of HIV-positive individuals from transmission, and theorize it may reduce risky sexual behaviour by the threat of punishment and encouraging socially desirable behaviour [37–39]. However, multiple U.S. studies that have shown living in a State with or without HIV specific criminalization laws does not affect sexual behaviour, condom use or disclosure, even among individuals aware of the law. [40–44]. Hence, there is no situation in which HIV specific criminalization laws actually decrease HIV transmission.

Furthermore, we hypothesize that even if there was an increase in condom use due to fear of prosecution, it would not match the effectiveness that ART has among HIV-positive aware individuals to reduce HIV transmission potential. This is even more pronounced within large urban centers in Canada, such as Toronto, where at the MLMC, where recruitment occurred for this study, 97% of HIV-positive MSM patients were on ART and of those on ART, 95% had an undetectable viral load [personal communication, Dr. F. Crouzat].

There are also great concerns surrounding how negative, crime-related framing of media reports and discourse surrounding HIV criminalization cases could deter HIV testing and increase HIV stigma and discrimination [1, 45]. Hence, HIV criminalization laws could also make disclosure and/or condom use conversations even harder. This is especially true among racialized and immigrant populations in Canada, who already face a burden of higher HIV prevalence within their communities, already have lower HIV testing rates, and have been overrepresented in the media and on criminal charges with regard to HIV criminalization cases [1, 46, 47].

There are some limitations to our study. First, we used a clinic-based recruitment and this could lead to selection bias with respect to MSM seeking primary care. It therefore may not be representative of all MSM in Toronto. We did not have sufficient variability in our outcome to be able to carry out a multivariable analysis. We may be missing some important confounders associated with both fear of prosecution and HIV testing behaviours.

Our flow chart models are based on the mathematical modeling and tree diagrams of other authors and have some limitations. First, some of the estimators we used came from external sources and may not be representative of our study. We also used disclosure rates at the time of HIV diagnosis, which in our study varied over the years from 1985–2011. Since disclosure rates may have changed significantly during this time period, we may have underestimated the disclosure rate. However, our disclosure rate of 72% was similar to the disclosure rate of 78.5% found in the O’Connell, Reed and Serovich, [23] which provided the models our flow charts models are based on. We also did not take into account all of the possible HIV prevention methods that could have decreased our transmission probability estimates, including serosorting, strategic positioning, circumcision, withdrawal, or the status of pre-exposure prophylaxis in the HIV-negative partner.

Our study was completed before the 2012 SCC non-disclosure decision. Therefore, a future study should be conducted to determine if the 2012 SCC decision has affected HIV testing in HIV-negative persons. Furthermore, future studies need to determine the awareness and knowledge of HIV criminalization laws among both HIV-positive and HIV-negative individuals because change in behaviour cannot occur if consequences of those behaviours are unknown. It is also unclear whether never having had a positive HIV test would be a legitimate argument against possible future prosecution. Commentators of the 2003 SCC decision (R v Williams) [48] have suggested that certain statements made by the SCC indicate an HIV-positive person who had yet to receive an official HIV-positive diagnosis still had an obligation to disclose their potential status to sexual partners and if they did not, they could be charge under the HIV criminalization laws [2, 49]. To date, there have been no criminal charges laid against any individuals who have not been formally diagnosed with HIV [2, 49].

## Conclusions

A minority of HIV-negative MSM receiving care at a primary care clinic in downtown Toronto reported being less likely to get an HIV test due to concern about prosecution. ART use by the HIV-positive partner is the most important factor in reducing HIV transmission in our model. When taking into account ART use by the HIV-positive partner, HIV-positive unaware individuals accounted for 66% of the HIV transmission potential. Reducing HIV testing by 7% increased the overall HIV transmission potential by 18.5% and the 23.7% of HIV-positive unaware group accounted for 73% of the total transmission potential. While the full impact of non-disclosure laws may still be unclear, decreasing the pool of individuals on ART through a reduction in HIV testing will not reduce HIV transmissions.
